# The Effectiveness of Active Rehabilitation Camp on Physical Performance of Disabled People Moving in Wheelchairs

**DOI:** 10.3390/ijerph18147572

**Published:** 2021-07-16

**Authors:** Anna Lipert, Kacper Wróbel, Michał Spychała, Paweł Rasmus, Dariusz Timler, Michał Marczak, Remigiusz Kozłowski

**Affiliations:** 1 Department of Sports Medicine, Medical University of Lodz, 92-213 Lodz, Poland; 2 Department of Management and Logistics in Healthcare, Medical University of Lodz, 90-131 Lodz, Poland; kacper.wrobel@stud.umed.lodz.pl (K.W.); michal.marczak@umed.lodz.pl (M.M.); 3 Faculty of Medicine, Medical University of Lodz, 92-213 Lodz, Poland; michal.spychala@stud.umed.lodz.pl; 4 Department of Medical Psychology, Medical University of Lodz, 90-131 Lodz, Poland; pawel.rasmus@umed.lodz.pl; 5 Department of Emergency Medicine and Disaster Medicine, Medical University of Lodz, 92-213 Lodz, Poland; dariusz.timler@umed.lodz.pl; 6 Center of Security Technologies in Logistics, Faculty of Management, University of Lodz, 90-237 Lodz, Poland; remigiusz.kozlowski@wz.uni.lodz.pl

**Keywords:** wheelchair users, training, active rehabilitation, disability

## Abstract

(1) Background: Regular participation in physical activity (PA) prevents many medical complications and improves the physical fitness of people with spinal cord injury, and in turn improves the functional independence, psychosocial status and quality of life. The goal of Active Rehabilitation Camps (ARCs) is to use various forms of PA in order for the participants to obtain the greatest efficiency and independence in everyday life. (2) Purpose: To evaluate the improvement in physical performance of people with chronic spinal cord disabilities moving in wheelchairs taking part in the Active Rehabilitation Camp depending on (a) sex, (b) type of disability, (c) the level of injury and (d) the type of wheelchair. (3) Methods: The study included 42 wheelchair users: 28 men and 14 women aged 18–65 years (34.7 ± 14.9 years) taking part in the Active Rehabilitation Camp. Finally, the study involved 27 paraplegics, 9 tetraplegics and 6 individuals with myelomeningocele. The participants took part in four fitness tests: (1) sprint test (SP)—individual time to cover a distance of 15 m in the wheelchair; (2) slalom test (SL)—time to ride between four cones front and back; (3) basketball ball throw at a distance (BT), (4) zig-zag test (ZZ)—riding continuously for 6 min on the designated track. The tests were performed at the beginning and at the end of the ACR. Active wheelchairs were used by 32 participants, and 10 participants used the classic wheelchairs. (4) Results: Paraplegics achieved the best average results in all the tests and the best improvement in physical performance in comparison to individuals with myelomeningocele and tetraplegics. People in active wheelchairs achieved a statistically significant improvement in the results of SL and ZZ (*p* < 0.001). People with injury above Th-9 level of the spinal cord achieved a statistically significant improvement in the results of SP (*p* < 0.01), SL and ZZ (*p* < 0.01). People with injury below Th6 achieved a statistically significant improvement (*p* < 0.05) in SP, SL and ZZ. (5) Conclusions: Regular PA during the Active Rehabilitation Camp improves the physical performance of disabled people in wheelchairs, but the scale of improvement of physical performance fitness depends on the type of wheelchair used and the level and the type of injury.

## 1. Introduction

About 10% of the global population, i.e., about 650 million people, have disabilities [1] and, of these, some 10% require a wheelchair [1]. Wheelchair users suffer injuries to the spinal cord, spinal nerves and cauda equina and/or have undergone lower limb amputation [1]. According to the International Standards for Neurological Classification for Spinal Injury [2], the anatomical site of the injury determines the categorization of the spinal cord injury (SCI). The two major categories are tetraplegia and paraplegia. Tetraplegia is identified as neural damage to cervical vertebrae one to seven, which produces impairments in both the upper and lower limbs as well as in the trunk, whereas paraplegia is identified with neural damage to the thoracic, lumbar or sacral vertebrae, precipitating trunk and lower limb dysfunction [2]. There are also different life-altering birth defects, e.g., myelomeningocele (MMC), leaving people with lifelong paralysis, incontinence and cognitive disabilities [2].

Wheelchair-bound individuals generally have a relatively inactive lifestyle [3] and show lower levels of physical fitness in comparison with the able-bodied population [4]. Research confirms that multiple dimensions of life, including increases in health concerns and healthcare burden, loss of functional independence, reduced participation in social, occupational and recreational opportunities or subsequent association with psychosocial sequelae, are affected when significant disability occurs [5]. Individuals moving in wheelchairs report high levels of limitation in both activities of daily living (ADLs) and instrumental ADLs (IADLs) [6]. A hypoactive lifestyle contributes to the development of secondary complications such as obesity, diabetes and cardiovascular disease [7,8]. In addition, a high association was reported between a sedentary lifestyle and metabolic syndrome among wheelchair users, increasing their risk of premature death [9]. Wheelchair users often experience severe depression, which produces social withdrawal [10] and lower quality of life [3]. Furthermore, prolonged sitting in wheelchair has been associated with an anterior pelvic tilt, tight hip flexors and lumbar lordosis, producing lower back pain [11]. There are several ways in which individuals using wheelchairs can remain active in daily life; however, some prerequisites should be met to enable them to have a more active lifestyle: the wheelchair should be optimally adjusted, and sport facilities should be easily accessible [12]. An active lifestyle often also requires a change in attitude or behavior [3,12].

The general objectives of rehabilitation for SCI patients include (1) to improve a patient’s independence in activities of daily living; (2) to help a patient accept a new lifestyle and (3) to aid a patient’s reintegration into society [13]. The rehabilitation process for individuals with SCI consists of three phases: acute, subacute and chronic [14]. The first two phases are part of the inpatients’ rehabilitation, which lasts from several weeks to several months, after which nearly 90% of SCI individuals return to their home and community [15]. Inpatient rehabilitation focuses on preventing SCI patients from secondary complications, promoting and enhancing neurorecovery, maximizing function and establishing optimal conditions for long-term maintenance of health and function [14]. During this initial rehabilitation, the implementation of training and interventions is concentrated to achieve an optimal level of wheelchair skill performance [16]. The chronic phase is realized usually during outpatient rehabilitation, which should be an ongoing, life-long plan to help prevent respiratory complications, maintain proper posture and mobility [17]. The optimal rehabilitation strategies for patients with SCI are difficult to define due to the challenges associated with rehabilitation research; these include a lack of standardization of interventions, therapeutic doses and outcome measures or multiple treatments that are prescribed by multiple healthcare professionals [14]. However, there is evidence that, no matter how specialized the acute care and inpatient rehabilitation for people with spinal cord injury, community programs have gained an increasingly important role in the subacute and long-term management of SCI [1]. The system of the Active Rehabilitation Camp (ARC) has been created in accordance with the Swedish concept from the late 1970s [18], which uses sport and physical recreation as a method of reconstructing the efficiency and self-dependence in day-to-day activities for motor-impaired individuals using wheelchairs. The ARC system provides intensive, goal-oriented, intentional, group-based, customized training and peer-support opportunities in a community environment for individuals with spinal cord injury [18]. It is a transfer of knowledge and practical life and social skills from experienced and active individuals with SCI (peer mentors) to newly injured individuals [18]. This community approach can be considered as a physical therapy treatment, which makes inpatient rehabilitation more meaningful and relevant to wheelchair individuals who report a number of unmet needs, especially related to psychological health, lifestyle, community functioning, self-efficacy and information [19,20]. Generally, there is a lack of research supporting trainings organized during ARC to improve physical function and aerobic fitness among wheelchair users with different motor impairments. One study indicated that the peer-based program AR can play an important role in promoting physical independence, wheelchair mobility and injury-management self-efficacy in community-dwelling individuals with SCI [18]. Therefore, the primary aim of the study was to evaluate the improvement in physical performance of people with chronic spinal cord disabilities moving in wheelchairs taking part in the Active Rehabilitation Camp. The secondary aim of the study was to determine the scale of differences in the improvement of wheelchair users’ physical performance depending on (a) sex, (b) type of disability, (c) the level of injury and (d) the type of wheelchair.

## 2. Material and Methods

### 2.1. The Study Design

The pre–post test prospective study involved 42 people aged 18–65 years moving on wheelchairs. The inclusive criteria were (1) the physical ability required for moving in a wheelchair; (2) a medical certificate on disability; (3) in the case of people after SCI, a period of neurological stabilization lasting about 6 months; (4) being cardio-respiratory efficient; (5) a medical certificate that there are no contraindications to participate in the Active Rehabilitation Camp and (6) active participation in the trainings during the training camp. The participants qualified for the study received written information about the study, the informed consent form to participate in the study and a questionnaire. The study was conducted during an Active Rehabilitation Camp organized in Poland by the Active Rehabilitation Foundation (FAR) in July 2016. The study was approved by the Institutional Review Board, approval Ref: RNN/286/15/KE/M.

### 2.2. Procedure

Firstly, the participants filled the application form disclosing personal data and information on the level of independence. Next, the fitness test was performed assessing the physical performance of the study participants at the beginning of the ARC.

Physical performance is determined by the development of specific skills and abilities to adapt to unexpected environmental influences and the reliable delivery of these skills and abilities in competitive situations [21]. Physical performance is affected by physiological capacities such as endurance, strength, speed or flexibility [22].

The fitness test consisted of four elements: (1) sprint test (SP)—individual timed ride over a distance of 15 m in the wheelchair; (2) slalom test (SL)—timed ride between four cones front and back; (3) basketball ball throw at a distance (BT), (4) zig-zag test (ZZ)—riding continuously for 6 min on the designated track. Individual agility tests, which were elements of the fitness test, were selected on the basis of previous research among people moving in wheelchairs [23,24]. During the next two weeks, the study participants took part in various activities within the ARC program. In the end of the ARC, the same fitness test was performed to assess the progress in the physical fitness of the study participants. Similar conditions were created at baseline and follow-up when the tests were performed. The measurements were taken two times for each estimated point, and the better result was taken into account.

### 2.3. The Active Rehabilitation Camp (ARC) Program

ARC program provides intensive, goal-oriented, intentional, group-based, customized training and peer-support opportunities for individuals with an SCI in a community environment. There are 10 key elements of the training camp: (1) peer mentors; (2) non-disabled assistants; (3) Activities of Daily Living (ADL) and wheelchair skills training; (4) use of sports and recreational activities; (5) education; (6) training environment; (7) admission criteria; (8) setting goals, initial and final assessment; (9) training of peer mentors and (10) duration of the AR camps [18]. The detail information about every element is as follows:

1. Peer mentors are experienced individuals with SCI who are involved in the training of participants as a real-life example of what participants could achieve.
2. Non-disabled assistants usually with a health professional education background support a group and are involved with organizational aspects, training aspects or with the provision of personal assistance to participants.
3. Training for ADL, such as training of toilet transfers, showering and dressing occurring at the natural time and environment involving custom-based ramps and stairs, is incorporated in the daily schedule with the help and under the guidance of peer mentors.
4. Sports and therapeutic recreation activities during ARC include a variety of sports and recreational activities. Most ARCs offer weightlifting and general fitness training, table tennis, swimming and archery. However, some of these sports may be excluded (e.g., swimming), and other sports may be included (e.g., basketball, self-defense) according to locally available equipment and facilities. Availability of the activity in the community usually influences the decision of whether to include the activity or not.
5. Education sessions may include introduction to SCI, wheelchair adjustments, prevention of pressure sores, prevention of urinary tract infections, bowel management and sexuality and fertility, and they are intended to help participants acquire or maintain knowledge that would allow them to optimally manage their condition.
6. Training environment means organizing the ARC in wheelchair friendly facilities (e.g., buildings with wheelchair access, accessible bathrooms, etc.) in hotels, schools, sports or recreational complexes, which creates a real-life learning environment.
7. Admission criteria ensure that the camp is suitable for the participants and minimize the level of risk, so the participants must be free of severe complications and be able to roll the wheelchair on a flat surface.
8. At the start of the ARC, participants are asked to provide background information for themselves and to complete a self-assessment related to their level of independence in ADL, their general condition and their training goals. This information is used to customize the intervention to their functional level and needs. Self-assessment occurring at the end of the ARC determines any changes in relation to outcomes.
9. Training of peer mentors means that the former participants of ARC can become peer mentors, which is possible after completing the special trainers’ training workshops that include a variety of topics, such as anatomy, prevention of complications, health promotion, management of impairments, role of the leader and organizational aspects.
10. Duration of the ARC usually varies from 5 to 10 days depending on available funding and personnel, but usually it is enough for participants to familiarize themselves with the program, to train and to interact with peer mentors and other participants.

However, the structure and content of the camp may vary between countries depending on culture and climate. Typically, camps have around 2–3 practical sessions during the day lasting between 1 and 3 h each. The practical sessions are designated to train wheelchair skills, strength or to practice a specific adapted sport such as table tennis, swimming, archery, wheelchair basketball and rugby, floorball and boccia. The aim of the sports activity is to increase the general physical fitness of the participants and positively influence their well-being. The team games teach cooperation in a group and shape social skills. Moreover, different sport activities provide the knowledge needed to consciously use various forms of physical activity and to motivate participants to continue working on themselves after the end of the camp. The ARC staff consists of instructors in wheelchairs and non-disabled people, who support the instructors in the efficient and safe conduct of classes and support the participants with their knowledge and experience acquired during the camps.

### 2.4. Statistical Analysis

Statistical analyses were performed using Statistical version 13.1 software (StatSoft, Tulsa, OK, USA). The Shapiro–Wilk test was used to check whether the data had a normal distribution. When data were normally distributed, the Student’s *t*-test was performed to analyze the differences between and inside the groups. When data were not normally distributed, the Mann–Whitney test was used to analyze the differences between the groups and the Wilcoxon test to analyze the differences inside the groups. If there were more than two groups to compare the results, the ANOVA test was used for the data with normal distribution or the Kruskal–Wallis test for the data with non-normal distribution. The effect size measure of differences between the results from the beginning and after 2 weeks of training was verified by Cohen’s d-test. It is defined as the difference between two means divided by a standard deviation for the data. Cohen classified effect sizes as small (d = 0.2), medium (d = 0.5) and large (d ≥ 0.8). All confidence intervals (CIs) are presented as 95% CI. Significant differences were accepted for all analyses at the level of *p* < 0.05.

## 3. Results

### 3.1. Characteristic of the Study Group

The general characteristic of the study group is shown in Table 1. Generally, the study participants were around 30 years old (34.7 ± 14.9 years), but the males were older than the females. The s males in the study had greater body mass than the females, but the average value of BMI indicated the correct body mass of the study participants. Over 64.3% of the study participants were people diagnosed with paraplegia. There were no females with tetraplegia, and none of the males were diagnosed with myelomeningocele core (Table 1).

### 3.2. Physical Performance before and after the Active Rehabilitation Camp of the Study Participants

**Figure 1:**
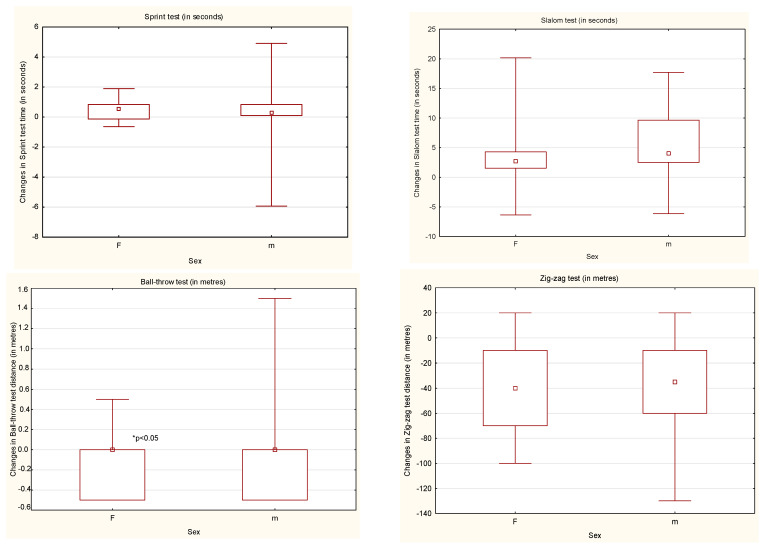
The changes in the results obtained before and after the ARC by the participants according to sex. F—female; m—male.

In all fitness tests in this study, the males obtained better results than the females with the same disability, but a statistically significant difference (*p* < 0.05) was noticed only in the ball-throw test results (Figure 1). After the Active Rehabilitation Camp (ARC), males significantly improved (*p* < 0.05) the results obtained in all the test. Females significantly improved (*p* < 0.05) the results obtained in three fitness tests except the ball-throw test (Table 2, Table 3, Table 4 and Table 5).

In general, after the ARC, progress in physical fitness test results was achieved by all the study participants. Participants with paraplegia achieved statistically significant (*p* < 0.05) progression in all the fitness tests. Participants with tetraplegia achieved a statistically significant progression in the slalom and zig-zag tests (*p* < 0.05). Participants with myelomeningocele core achieved a statistically significant progression in the zig-zag test (*p* < 0.05) (Table 2, Table 3, Table 4 and Table 5).

**Figure 2:**
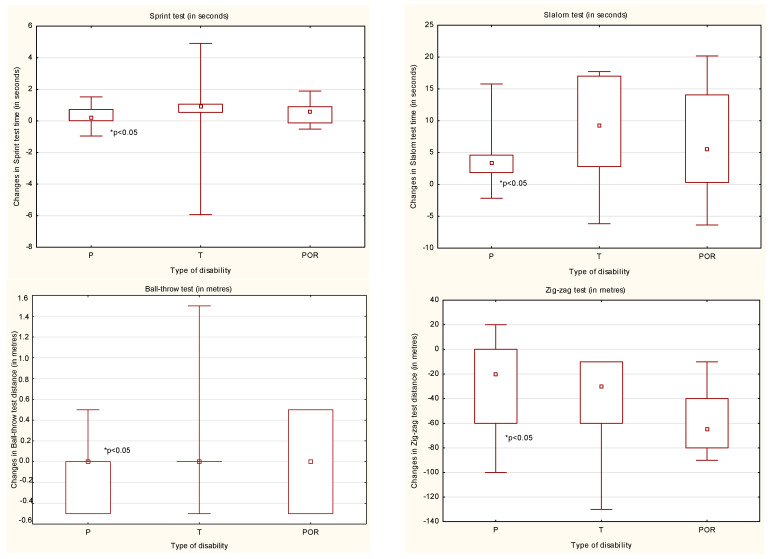
The changes in the results obtained before and after the ARC by the participants according to the type of injury. P—paraplegics; T—tetraplegics; POR—people with myelomeningocele core.

The best results in all fitness tests were obtained by people with paraplegia. The results from all the fitness tests were statistically significantly better (*p* < 0.05) in comparison to those of people with tetraplegia (Figure 2). Comparing people with paraplegia to those with myelomeningocele core, statistically significantly better results (*p* < 0.05) were obtained in three from four fitness tests (Figure 2). People with myelomeningocele core obtained better results in all the tests in comparison to people with tetraplegia, but the difference was statistically significant (*p* < 0.05) only in the zig-zag test (Figure 2).

**Figure 3:**
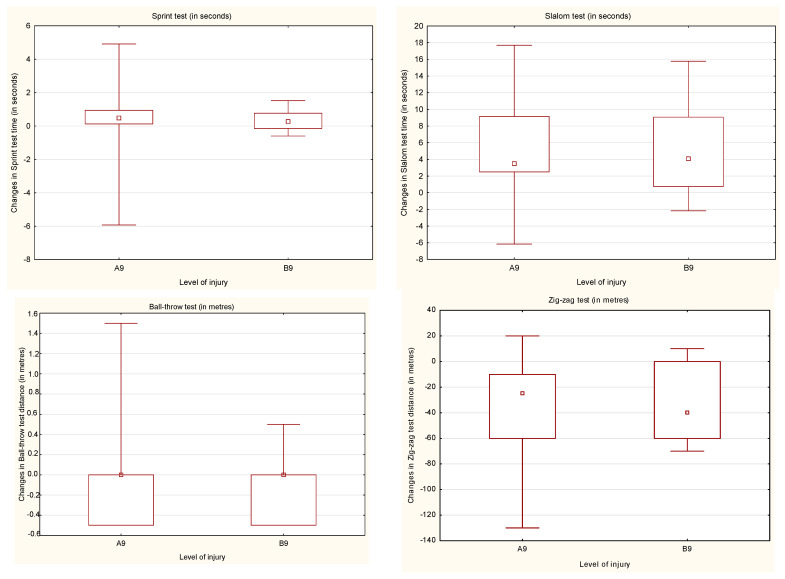
The changes in the results obtained before and after the ARC by the participants according to the level of injury. A9—above Th-9; B9—below Th-9.

After the ARC, people with spinal cord injury below Th-9 improved their results in all the tests with statistically significant difference of the results in sprint, slalom and zig-zag tests (*p* < 0.05). People with spinal cord injury at the level of Th-9 and above also improved their results in sprint, slalom and zig-zag tests with statistically significant differences in all of the tests (*p* < 0.05) (Table 2, Table 3, Table 4 and Table 5, Figure 3).

**Figure 4:**
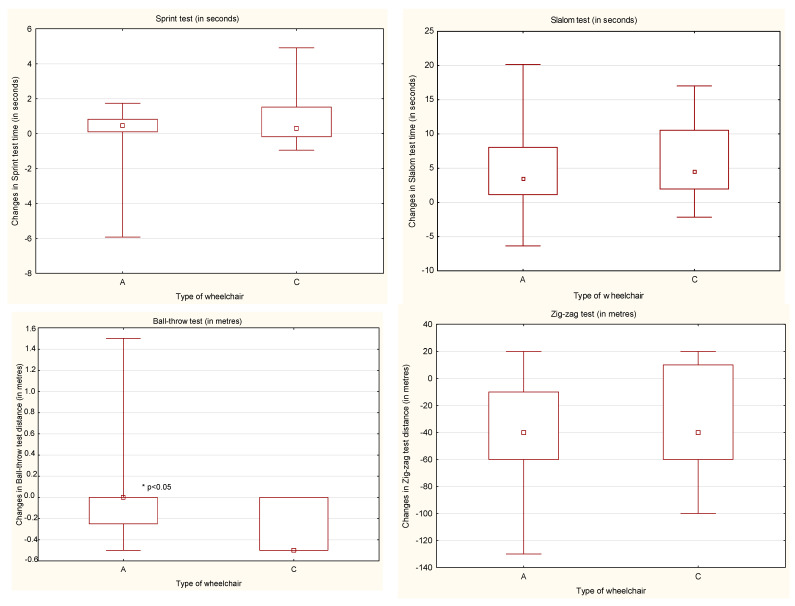
The changes in the results obtained before and after the ARC by the participants according to the type of wheelchair. A—active wheelchair; C—classic wheelchair.

Generally, people using active wheelchairs during fitness tests had better results than people using classical wheelchairs (Figure 4). After the ARC, people using active wheelchairs achieved progress in their physical fitness, which was noticed during all the tests and statistically significant differences (*p* < 0.05) in the results were obtained in the sprint, slalom and zig-zag tests. People using the classic wheelchairs also improved their physical fitness in all fitness tests with statistically significant difference (*p* < 0.05) in the results of the slalom, ball-throw and zig-zag tests (Table 2, Table 3, Table 4 and Table 5).

## 4. Discussion

The physical performance of people moving in wheelchairs is generally worse compared to that of able-bodied individuals mainly due to the impaired muscle function in the lower limbs and an inactive lifestyle [25]. It seems reasonable to create challenging sport events as part of a rehabilitation process, which helps those people initiate or keep training to improve physical fitness and health. The presented study showed that the Active Rehabilitation Camp positively influenced all the participants, who achieved an improvement in their physical performance irrespective of the type of their spinal cord disability. The obtained results will fill the gap in the current knowledge about physical performance and insufficient evidence regarding the effects of exercise, or specific types of exercise, on any of the fitness components [26] of unable-bodied people moving in wheelchairs especially those with long-term SCI (>10 years) [27]. There is also a high need for quality studies assessing the effectiveness, cost efficiency and the perceived benefits of the ARC approach [18].

Measuring improvement in physical performance including tests of wheelchair skills and propulsion can be translated into increased independence and quality of life for people with spinal cord injury [26]. Although, the improvement of physical performance was observed in all of the study participants, the spectrum of progress was not similar for all of the wheelchair users and depended on different variables.

Generally, the males in the study obtained better results than the female study participants with the same disability, but this is in line with natural biological preference. Males were stronger especially in the ball-throw test, which assesses the performance of explosive force and so requires good muscle strength. Muscle strength is a highly relevant fitness outcome in the chronic SCI population, as improvements in strength have a significant impact on the ability to perform activities of daily living [26]. The research analyzing the effects of progressive resistance exercises (PREs) and endurance exercises (EEs) on upper extremity in patients with paraplegia [28,29] confirms its importance in increasing activities of daily living (ADLs) in patients with spinal cord injury (SCI).

The best results were obtained by people with paraplegia. The slowest progress in physical performance was observed in tetraplegics. However, people with tetraplegia and those with paraplegia have a different change of fitness, so it is always recommended to study these groups separately [30]. In addition, time since injury (TSI) seems to have an effect on the fitness of people with paraplegia in contrast to those with tetraplegia [27]. It was noticed that the physical capacity of people with a paraplegia is significantly associated with TSI and the lowest is observed among those with the longest TSI [31]. It seems that wheelchair-specific fitness can diminish over time after the injury, so it is important to pay special attention to the group with a long TSI (>30 years) to maintain their long-term fitness. However, in the analyzed group, the average TSI for paraplegics was below 5 years and for tetraplegics it was two years shorter, so the obtained progress in the results was not dependent on it [27,31]. Being physically active in everyday life also seemed to be positively related to wheelchair-specific fitness in persons with tetraplegia, but not in those with paraplegia [27]. It was also confirmed that resistance training improved health, flexibility and strength in wheelchair-bound nursing home residents, and a lack of physical activity in everyday life may increase dependence on others [32,33,34]. Therefore, the improvement of physical performance among the study participants was possible because of the active participation in everyday activities organized during the camp as most of the participants reported an inactive lifestyle.

The level of SCI was observed to be one of the most important factors influencing physical work capacity in wheelchair-dependent paraplegics [35]. The same dependence was noticed in the present study where people with spinal cord injury below Th-9 achieved better improvement of their physical performance in all the tests in comparison to people with SCI at the height of Th-9 and above. However, it should be remembered that some changes in functional efficiency of the ARC participants might be not directly related to the improvement of their physical performance, but from the time devoted to shaping selected motor skills. Brurok et al. noticed that only five weeks of intensive rehabilitation organized in 30-min-long training sessions improved the physical work capacity of individuals with spinal cord injury [36].

The differences in physical performance improvement were also analyzed depending on the wheelchair that was being used during the camp. Active wheelchairs have made it possible for participants to achieve better results in the tests than classical wheelchairs. It was possible because sport wheelchairs have undergone ergonomic modifications that improve the biomechanics of the user and adherence to physical activity programs [10]. The importance of wheelchair fit has long been described, and the need to ensure a proper match between the equipment and wheelchair user has been highlighted [37,38]. Although, there is little research to compare the obtained results, it seems that the greater progress was obtained by the study participants using the active wheelchairs. The results of the Andrews study confirmed that the wheelchair type influence the propulsion technique [39] and so it can influence the performance improvement.

There are a few strengths of the present study. To our knowledge, there is little and research [18] assessing the efficiency of Active Rehabilitation Camps in improving the physical performance of people in wheelchairs, so the study is unique. It was a pre–post test prospective study, in which the physical performance was assessed objectively with special fitness tests adapted to the needs of people with disabilities. However, some limitations should be emphasized. The main limitation of the present study was a small sample size, and therefore, more extensive research is required that would confirm the obtained results. However, this limitation is specific for studies concerning people with disabilities due to the accessibility to disabled people. It is also difficult to ensure a very homogeneous group, due to the fact that there are many reasons that cause a person to be disabled and require a wheelchair. At the same time, it is not possible to prevent disabled people from participating in Active Rehabilitation Camps only because of the conducted research and the need to gather people with similar disabilities. In future studies, it would be also interesting to see how much the camp improves the independence in activities of daily living and if this correlation exists.

Although the ARC has been widely organized internationally, it has received little to no attention in the literature [18]. However, there is compelling evidence that, even with well-organized acute care and inpatient rehabilitation, newly injured individuals feel unprepared physically and psychologically to transition to home [19,40]. Moreover, people with spinal cord or the other disabilities requiring them to move on wheelchair are characterized by a low level of physical activity, so they should be provided with different opportunities to maintain a physically active lifestyle [10]. In that context, community programs organized according to the approach of the Active Rehabilitation Camp and addressing the long-term physical, emotional, independent living and lifestyle needs of wheelchair users would be beneficial in the subacute and long-term management of physically impaired people [41]. It should be also underlined that the rehabilitation process begun during the ARC should be continued, and the learned skills have to be consolidated and developed further, because in people with spinal cord injury using wheelchairs a lack of physical activity leads to losing independence in everyday life [3].

## 5. Conclusions

The results show that participation in the Active Rehabilitation Camp by wheelchair users improves their physical performance, which may help them obtain greater independence in everyday life. However, the scale of improvement of physical performance depends on the type of applied wheelchair and the level and the type of injury. This is one of few studies analyzing the ARC approach, and it provides the basis for further studies exploring its effectiveness. It could also potentially support higher integration in the healthcare system and long-term funding of these kind of programs.

## Figures and Tables

**Table 1:** The general characteristic of the study group (*n* = 42).

	All the Study Group (*n* = 42)	Paraplegics (*n* = 27)	Tetraplegics (*n* = 9)	People with Myelomeningocele Core (*n* = 6)
	Mean (±SD)	Mean (±SD)	Mean (±SD)	Mean (±SD)
Age (years)	34.7 ± 14.9	35.4 ± 14.3	39.0 ± 18.9	25.0 ± 6.0
Body mass (kg)	67.0 ± 15.0	71.2 ± 11.5	70.6 ± 8.0	42.5 ± 14.4
Height (cm)	170.6 ± 14.7	173.5 ± 8.0	181.3 ± 6.6	141.2 ± 9.2
Body mass index (kg/m^2^)	22.8 ± 3.9	23.6 ± 3.4	21.5 ± 2.7	21.2 ± 6.5
Time since injury (TSI)	6.8 ± 10.3	4.2 ± 8.4	2.3 ± 1.9	29.0 ± 6.0
Habitual PA	N (%)	N (%)	N (%)	N (%)
Yes	3 (7.2)	1 (3.7)	1 (11.1)	1 (16.7)
No	39 (92.8)	26 (96.3)	8 (88.9)	5 (83.3)
	**All the Study Group (*n* = 42)**		**Males (*n* = 28)**	**Females (*n* = 14)**
	Mean (±SD)		Mean (±SD)	Mean (±SD)
Age (years)	34.7 ± 14.9		37.6 ± 14.6	28.9 ± 14.2
Body mass (kg)	67.0 ± 15.0		73.2 ± 8.6	54.6 ± 17.5
Height (cm)	170.6 ± 14.7		178.1 ± 7.4	155.5 ± 14.3
Body mass index (kg/m^2^)	22.8 ± 3.9		23.1 ± 2.7	22.3 ± 5.7
Time since injury (TSI) (in years)	6.8 ± 10.3		3.4 ± 7.3	13.5 ± 12.4
	N (%)		N (%)	N (%)
Type of disability				
Paraplegia	27 (64.3)		19 (67.9)	8 (57.1)
Tetraplegia	9 (21.4)		9 (32.1)	0 (0)
Myelomeningocele core	6 (14.3)		0 (0)	6 (42.9)
Habitual PA				
Yes	3 (7.2)		1 (3.6)	2 (14.3)
No	39 (92.8)		27 (96.4)	12 (85.7)

**Table 2:** The results obtained in the sprint test by the study participants before and after ARC.

Sprint Test (Time in Seconds)	Before ARC	After ARC	Change	*p* Value	d
	Mean (95% CI)				
Sex					
Males	7.11 (7.27; 10.46)	6.76 (7.02; 10.03)	0.26 (−0.28; 0.96)	0.005	0.28
Females	8.23 (6.99; 11.08)	7.50 (6.35; 10.74)	0.57 (0.07; 0.91)	0.030	0.33
Type of disability					
Paraplegics	6.68 (6.53; 8.03)	6.38 (6.20; 7.76)	0.22 (0.07; 0.53)	0.012	0
Tetraplegics	12.13 (9.52; 16.96)	11.08 (9.25; 16.12)	0.94 (−1.61; 2.72)	0.110	0.25
POR	8.60 (5.28; 14.38)	8.22 (4.31; 14.25)	0.56 (−0.34; 1.43)	0.173	0.09
Level of injury					
Below Th-9	6.35 (6.14; 7.96)	6.06 (5.84; 7.49)	0.26 (0.04;0.73)	0.035	0.21
Above Th-9	8.41 (7.88; 11.85)	8.15 (7.62; 11.41)	0.47 (−0.44; 1.15)	0.013	0.07
Type of wheelchair					
Active	7.58 (7.45; 9.70)	7.01 (7.01; 9.62)	0.46 (−0.19; 0.71)	0.001	0.15
Classic	7.63 (5.86; 14.18)	7.36 (5.97; 12.48)	0.30 (−0.41; 2.00)	0.169	0.07

**Table 3:** The results obtained in the slalom test by the study participants before and after ARC.

Slalom Test (Time in Seconds)	Before ARC	After ARC	Change	*p* Value	d
	Mean (95% CI)				
Sex					
Males	31.06 (31.18; 48.29)	28.50 (27.04; 40.97)	4.03 (3.33; 8.14)	<0.001	0.15
Females	31.24 (27.64; 50.01)	29.53 (25.31; 43.99)	2.71 (0.52; 7.84)	0.009	0.11
Type of disability					
Paraplegics	29.28 (26.58; 36.05)	25.49 (22.77; 31.63)	3.37 (2.56; 5.69)	<0.001	0.36
Tetraplegics	50.67 (37.41; 81.76)	46.65 (35.34; 68.61)	9.22 (0.78; 14.43)	0.051	0.16
POR	43.17 (24.86; 66.59)	42.84 (25.96; 52.39)	5.56 (−3.44; 16.54)	0.116	0.06
Level of injury					
Below Th-9	28.57 (25.07; 35.92)	23.86 (21.87; 29.75)	4.08 (1.72; 7.65)	0.005	0.65
Above Th-9	32.62 (32.29; 54.52)	31.17 (28.92; 47.51)	3.48 (2.50; 7.88)	0.001	0.22
Type of wheelchair					
Active	29.35 (29.92; 43.42)	27.02 (26.56; 37.15)	3.48 (2.53; 7.09)	<0.001	0.12
Classic	35.64 (29.62; 66.94)	32.08 (25.46; 58.10)	4.44 (2.21; 10.80)	0.012	0.12

**Table 4:** The results obtained in the ball-throw test by the study participants before and after ARC.

Ball-Throw Test (Distance in Meters)	Before ARC	After ARC	Change	*p* Value	d
	Mean (95% CI)				
Sex					
Males	6.00 (4.61; 6.78)	6.00 (4.66; 6.95)	0.00 (−0.27; 0.05)	0.010	0
Females	4.75 (3.54; 5.32)	4.75 (3.58; 5.35)	0.00 (−0.25; 0.17)	0.735	0
Type of disability					
Paraplegics	6.50 (5.98; 7.39)	6.50 (5.98; 7.59)	0.00 (−0.29; −0.04)	0.028	0
Tetraplegics	2.50 (1.35; 3.31)	2.50 (1.45; 2.99)	0.00 (−0.31; 0.53)	0.655	0
POR	3.00 (2.06; 4.60)	2.75 (1.90; 4.77)	0.00 (−0.47; 0.47)	1.00	1.00
Level of injury					
Below Th-9	7.25 (6.06; 8.29)	7.00 (6.06; 8.51)	0.00 (−0.31; 0.09)	0.310	0.10
Above Th-9	5.00 (3.53; 5.64)	5.25 (3.59; 5.77)	0.00 (−0.28; 0.10)	0.161	0.04
Type of wheelchair					
Active	5.50 (4.41; 6.15)	5.75 (4.38; 6.21)	0.00 (−0.16; 0.13)	0.649	0.08
Classic	5.50 (3.13; 7.36)	5.75 (3.38; 7.72)	−0.50 (−0.48; −0.11)	0.028	0.07

**Table 5:** The results obtained in the zig-zag test by the study participants before and after ARC.

Zig-Zag Test (Distance in Meters)	Before ARC	After ARC	Change	*p* Value	d
	Mean (95% CI)				
Sex					
Males	415.00 (324.09; 433.77)	425.00 (361.18; 467.39)	0.00 (−0.27; 0.05)	<0.001	0.07
Females	400.00 (314.61; 461.11)	440.00 (347.63; 505.22)	0.00 (−0.25; 0.17)	0.008	0.30
Type of disability					
Paraplegics	460.00 (397.34; 475.99)	500.00 (424.11; 508.48)	−20.00 (−43.79; −15.47)	0.001	0.39
Tetraplegics	170.00 (153.81; 277.30)	200.00 (187.67; 327.89)	−30.00 (−71.69; −12.76)	0.008	0.35
POR	390.00 (228.39; 541.61)	440.00 (302.59; 584.08)	−65.00 (−89.05; −27.62)	0.028	0.35
Level of injury					
Below Th-9	475.00 (429.13; 513.73)	520.00 (451.75; 549.68)	−40.00 (−46.45; −12.12)	0.011	0.57
Above Th-9	300.00 (264.20; 383.98)	365.00 (298.62; 419.56)	−25.00 (−52.93; −17.07)	0.001	0.48
Type of wheelchair					
Active	435.00 (347.00; 483.03)	465.00 (384.47; 483.03)	−40.00 (−49.34; −25.03)	<0.001	0.22
Classic	335.00 (247.24; 422.76)	370.00 (280.13; 457.87)	−40.00 (−66.38; −1.62)	0.083	0.28

## Data Availability

The datasets used and/or analyzed during the current study are available from the corresponding author on reasonable request.

## Correction Statement

The Editorial Office and Editorial Board would like to inform readers of a post-publication review process that was conducted related to this publication. Following concerns about a potential conflict of interest between the authors and the original Academic Editor, the Editorial Office has conducted a post-publication peer-review of this article. This process included the recruitment of a new Academic Editor to ensure full compliance with MDPI’s Editorial Process (https://www.mdpi.com/editorial_process). As a result of this process, the original Academic Editor was removed, and a new Academic Editor is listed. The new Academic Editor is satisfied that the editorial process for this article has been completed in accordance with MDPI’s Editorial Process policy.
